# “H”‐like Organic Nanowire Heterojunctions Constructed from Cooperative Molecular Assembly for Photonic Applications

**DOI:** 10.1002/advs.201500130

**Published:** 2015-07-14

**Authors:** Wei Yao, Guangchao Han, Fu Huang, Manman Chu, Qian Peng, Fengqin Hu, Yuanping Yi, Hua Jiang, Jiannian Yao, Yong Sheng Zhao

**Affiliations:** ^1^ Beijing National Laboratory for Molecular Sciences (BNLMS)Institute of ChemistryChinese Academy of SciencesBeijing100190P.R. China; ^2^ College of ChemistryBeijing Normal UniversityBeijing100875P.R. China

**Keywords:** energy transfer, molecular assembly, nanowire heterojunction, organic nanomaterial, photoswitch nanophotonics

## Abstract

“**H**”**‐like organic nanowire heterojunctions** with two parallel 2‐acetyl‐6‐dimethylamino‐naphthalene wires vertically bridged by one 2,4,5‐triphenylimidazole wire are prepared via cooperative molecular assembly in liquid phase. The exciton conversion at the junction interfaces is beneficial for the design of multichannel light‐controlled photo­switches. The results provide better understanding of molecular assembly toward specific structures and open up new prospects for the creation of novel photonic materials.

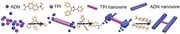

Controlling the optical parameters (e.g., intensity, wavelength, polarization, and phase) of the light outcoupling, is key to the development of integrated photonic elements toward the next‐generation optical computing platforms. 1D nanomaterials, acting as building blocks in miniaturized optoelectronics,^1^ have attracted great research interests for manipulating photons at micro/nanoscale. Assembling 1D nanowires into complex heterostructures with high spatial and angular precision is critical for realizing various functional devices, including photonic routers,^2^ logic gates,^3^ modulators,^4^ wavelength filters,^5^ and multiplexers.^6^ So far, most composite nanowire structures were fabricated with lithography or accurate mechanical micromanipulation;^7^ however, these methods are either too complicated or unfit for the achievement of stable heterojunctions due to the simple point contact between the different nanowires. One alternative route is to construct chemical heterogeneous structures with partially embedded and stable connection via a bottom‐up self‐assembly strategy.^8^

Organic molecules are promising candidates for designing and constructing various heterostructures toward photonic applications, due to their ease of structure construction,^9^ broad spectral tunability^10^ and self‐assembly characteristics for bottom‐up fabrication.^11^ Small organic molecules tend to form single component crystalline structures through an assembly process driven by the weak interactions among the molecules, such as hydrogen bonds, van der Waals forces, π–π interactions, etc.^12^ Organic composite nanomaterials with specific structures and compositions would be achieved via rational molecular design to tune the intermolecular interactions.^13^ The exciton conversion between different components with matched energy levels at the junctions of the heterostructures can be utilized to finely tailor the spatial excitonic emission along the path of light propagation,^14^ which offers an effective way to manipulate the light signals and realize functional devices. However, the lack of understanding of molecular coassembly has severely hampered the construction of organic multicomponent heterostructures.

Herein, we report the construction of an “H”‐like organic nanowire heterojunction for photonic modulation purposes through the coassembly of two rational designed components in liquid phase. The cooperative molecular assembly mechanism of the “H”‐like heterostructure has been elaborated from the on‐site observation of the assembly process combined with the theoretical calculation of the molecule binding energies. One compound self‐assemble into 1D nanowires first and the two ends of each wire act as nucleation centers for the site‐specific epitaxial growth of the other component, resulting in the “H”‐like nanowire heterostructures with partially embedded junctions. These stable junctions showed effective energy transfer from the bridge wire (energy donor) to the side wires (energy acceptor). By adjusting the population of photogenerated donor excitons, we modulated the output signal of accepter to realize “on” and “off” states, which can be used for optical switch in miniaturized photonic circuits. These results offer a comprehensive understanding of the coassembly mechanism of organic composite materials and open a new way to design and construct miniaturized photonic devices.

Nanowire heterojunctions may be controllably constructed if the intermolecular interactions along the growth direction are properly designed between the two different molecules. In the previous work, we found that 2,4,5‐triphenylimidazole (TPI) molecules (**Figure** **1**a) can form 1D nanostructures via the intermolecular hydrogen bonding interactions^15^ and 2‐acetyl‐6‐dimethylamino‐naphthalene (ADN) molecules (Figure 1a) can self‐assemble into nanowires from π–π stacking[[qv: 9a]] (Figure S1, Supporting Information). As illustrated in Figure 1b, the simulated optimal steric configuration reveals that there is a hydrogen bonding interaction between TPI and ADN molecules. Induced by the hydrogen bonding, one compound might nucleate onto the pregrown structure of the other and grow into nanowire heterostructures during the subsequent assembly. Moreover, there is a good spectroscopic overlap between the emission of TPI and the absorption of ADN, which would facilitate an efficient FRET and exciton conversion from TPI to ADN (Figure S2, Supporting Information).

**Figure 1:**
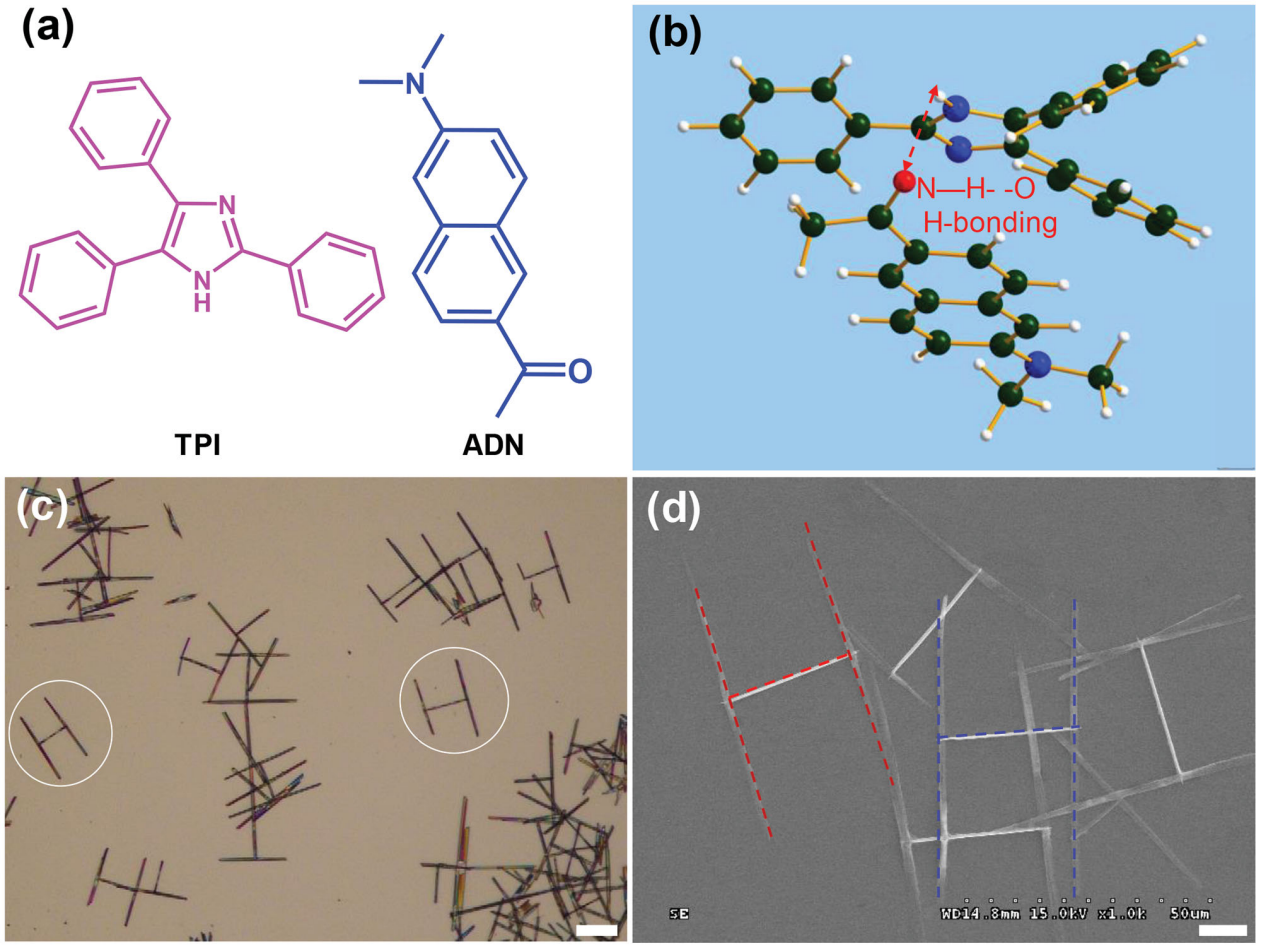
a) Molecular structures of TPI and ADN. b) The simulated optimal steric configuration of TPI and ADN molecules, which shows the intermolecular hydrogen bonding interactions between them. c) Bright‐field optical microscopy image of the “H”‐like organic nanowire heterojunctions. Scale bar is 20 μm. d) SEM image of the “H”‐like heterostructures. The red‐ and blue dashed lines are added to identify the single “H”‐like structures. Scale bar is 10 μm.

We adopted TPI and ADN as the model compounds to construct binary organic nanowire heterostructures via cooperative molecular assembly. In a typical preparation, hot ultrapure water (80 °C) as a poor solvent was added dropwise into 100 μL of the mixed solution of TPI and ADN in THF to induce the nucleation and growth of them (see the Experimental Section for details). Figure 1c displays the bright‐field optical microscopy image of the as‐prepared structures deposited on glass. It can be seen that almost all of the obtained aggregates are “H”‐like nanowire heterojunctions with two parallel wires bridged vertically by another wire. The typical scanning electron microscopy (SEM) image (Figure 1d) of the obtained “H”‐like heterojunctions shows that the average diameter and length of the bridge nanowires are 200 nm and 30 μm, respectively, while the side nanowires have a uniform diameter of about 500 nm and length up to 70 μm. By changing the concentration ratios of TPI/ADN, the size of the side nanowires in the heterostructures can be effectively tuned from 20 to 130 μm (Figure S3, Supporting Information).

The fluorescence emissions at different positions of the “H”‐structure were characterized to determine the structural composition of the “H”‐like nanowire heterostructures. **Figure** **2**a displays the bright‐field optical microscopy image of a typical “H” heterojunction consisting of two parallel nanowires (marked as NW 1 and NW 3) and another nanowire that is perpendicular to them (NW 2). Each part of the heterostructure was excited with a focused laser beam (351 nm, Figure 2b), and the corresponding spatially resolved microarea PL spectra were revealed in Figure 2c. The PL spectra recorded from the NW 1 and NW 3 present maximum emission at 450 nm, which are in good consistence with that of the ADN nanowires.[[qv: 9a]] Meanwhile, the PL spectrum of the NW 2 presents a peak centered at 405 nm, which is in accordance with that of the TPI nanowires.^15^ These results suggest that the bridged nanowire of “H”‐like organic nanowire heterojunctions is composed of TPI, while the two parallel nanowires are assembled from ADN molecules.

**Figure 2:**
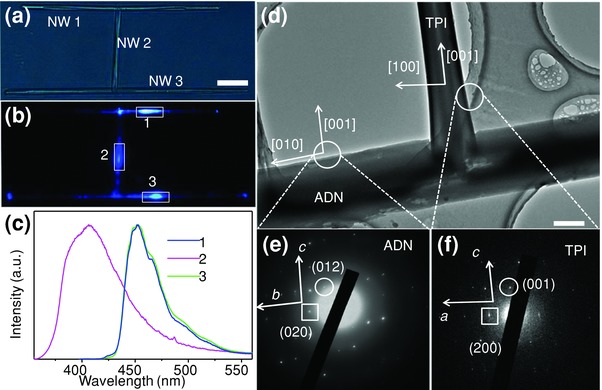
a) Bright‐field and b) PL microscopy images obtained from a single “H”‐like nanowire heterojunction by exciting the nanowire heterostructures at different positions. Scale bar is 10 μm. c) Spatially resolved PL spectra collected from the three different excitation spots as shown in (b). d) TEM image of a single heterostructure at the junction part. Scale bar is 500 nm. SAED patterns of the e) ADN and f) TPI nanowires taken from the selected areas in (d).

The molecular orientation and arrangement in these “H”‐like heterostructures were determined using transmission electron microscopy (TEM) and selected‐area electron diffraction (SAED) measurements. Figure 2d shows a typical TEM image of the junction part of an “H”‐like nanowire heterostructure, revealing that the TPI and ADN nanowires have regular morphologies and smooth surfaces. The end of the TPI wire was embedded in the ADN wire and the interface between them can be clearly identified. The SAED patterns in Figure 2e,f indicate that both the ADN and the TPI nanowires are single‐crystalline, and the growth direction is matched with the *b*‐axis of ADN crystal (CCDC‐932983), and the *c*‐axis of TPI crystal (CCDC No. 720571), respectively. These matched results further confirm the components of the two parallel nanowires (ADN) and the bridged nanowire (TPI) in the heterostructures.

We monitored the formation process of the “H”‐like heterostructures in‐situ (see Video S1, Supporting Information) to investigate the mechanism of the cooperative molecular assembly. From the recorded video, we can see that there are only TPI nanowires formed in the solution at the initial stage. A few seconds later, the ADN molecules start to appear at the ends of TPI wires, and continue to grow epitaxially along the direction perpendicular to the preformed TPI nanowires. With the progress of the growth of ADN, “H”‐like nanowire heterojunctions with uniform dimensions were finally obtained and dispersed in the solutions. The bright‐field microscopy images in **Figure** **3**a–d show the temporal morphologies of a single heterostructure monitored on‐site during the assembly process. The evolution of the heterojunction from 5 to 80 s after the dropping of water further testifies that the TPI nanowires formed first, and then the two ends of the as grown wires act as the site‐specific nucleation centers for the heteroepitaxial growth of ADN nanowires, resulting in the formation of unique “H” heterostructures with two parallel ADN nanowires bridged vertically by one TPI nanowire.

**Figure 3:**
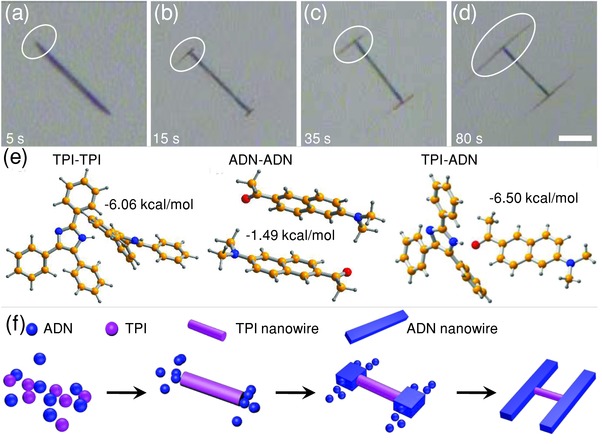
Temporal bright‐field optical microscopy images at different growth stages taken at a) 5 s, b) 15 s, c) 35 s, and d) 80 s after the dropping of water into the mixed solution. Scale bar is 10 μm. e) The configurations of TPI–TPI along [001] direction, ADN–ADN along [010] direction, and TPI–ADN at the junctions. f) Schematic illustration for the growth process of the “H”‐like nanowire heterojunctions.

This cooperative assembly process of the “H”‐like organic nanowire heterojunctions is pertinent to the intermolecular interactions. We calculated the binding energy of TPI–TPI, ADN–ADN, and TPI–ADN to evaluate the intermolecular interactions during self‐assembly. Figure 3e shows the steric configuration of TPI–TPI dimer along the TPI growth direction [001] (left), ADN–ADN dimer along assembly direction [010] (middle) and TPI–ADN configuration at the junction region (right). The calculated binding energy of the TPI–TPI configuration is as high as −6.06 kcal mol^−1^ due to the strong hydrogen bonding interactions, while the ADN–ADN has a weaker binding energy of −1.49 kcal mol^−1^. Combining the SAED results with the crystal structures of ADN and TPI, we can confirm the molecule arrangement at the junction region (Figure S4, Supporting Information) and construct a TPI–ADN molecular organization configurations (Figure 3e, right). The hydrogen bonding with a distance of 1.94 Å results in a binding energy of −6.50 kcal mol^−1^. Because one TPI molecule can form double hydrogen bonds with two neighboring molecules, the intermolecular interaction strengths between TPI–TPI are larger than TPI–ADN. As a result, the difference in the intermolecular interaction strengths, TPI–TPI (hydrogen bonds) > TPI–ADN (hydrogen bonds) > ADN–ADN (π–π stacking), leads to selective nucleation of ADN onto the ends of the preformed TPI nanostructures.

Based on the observation and discussion above, we propose an optimized model for the formation process of the “H”‐like nanowire heterojunctions, as illustrated in Figure 3f. At the initial stage, TPI molecules in the mixed solution (with the strongest intermolecular interaction) aggregated first to form 1D nanowires along the *c‐*axis under the driving force of hydrogen bonding. The —NH groups are exposed from the end facets of the single‐crystalline TPI nanowires, which would induce the selective nucleation of ADN molecules via the hydrogen bonding interaction between the TPI and ADN molecules. Note that TPI–ADN has a stronger binding than that of ADN–ADN, and the TPI end facets have higher surface energies; therefore, the ADN molecules selectively interact with the exposed TPI molecules at the tips of the TPI nanowires and nucleated there, before their epitaxial growth via π–π stacking along the direction perpendicular to the TPI nanowires to form the final “H”‐like heterojunctions. Our results well demonstrated the assembly processes from deliberately selected small molecules to desired composite nanostructures, which would potentially serve as a guideline for the construction of complex heterostructures at micro/nanoscale toward specific electronic and photonic applications.

In organic active waveguides, the strong coupling between photons and excitons results in the formation of exciton polaritons (EPs),^16^ which can propagate toward the heterojunction interface and drastically alter the light transmission.^17^ The spatial excitonic emission along the path of light propagation can be finely engineered through the donor–acceptor energy transfer in the organic heterostructures (**Figure** **4**a). The “H”‐like heterostructures offers a model system for demonstrating that the output from the junctions can be actively controlled by modulating the excitons conversion. As illustrated in Figure 4a, TPI molecules can be pumped to singlet excited states by UV radiation with a large energy gap of ≈3.1 eV, thereby, TPI excitons can transfer the energy to ADN at the junction interfaces via dipole–dipole interaction. The exciton conversion provides an effective way to modulate the excitonic and photonic processes in the ADN phase by controllably pump the TPI part in the heterostructure.

**Figure 4:**
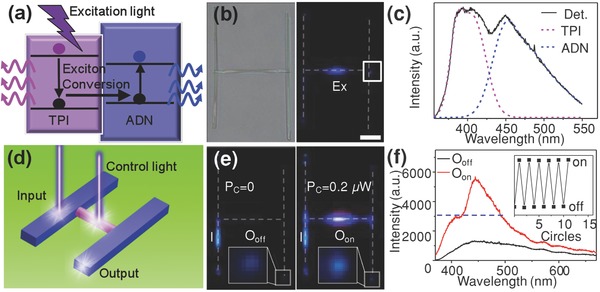
a) Schematic of the exciton conversion from TPI to ADN at the junction interface. b) Bright‐field and PL microscopy images obtained from a single “H”‐like heterostructure recorded by exciting the TPI nanowire with a focused laser. Ex: the excitation spot. Scale bar is 10 μm. c) The corresponding PL spectrum collected from the junction part marked with the white square in (b). The spectrum can be fitted to the emissions of the TPI and ADN nanowires. d) Schematic illustration for the design of a light‐controlled photoswitch. The output optical signal can be modulated by changing the control laser based on the exciton conversion from TPI to ADN. e) PL microscopy images of the “H”‐like heterostructure excited with a constant input laser power (*P*_I_ = 0.2 μW) and a control power of 0 (left), 0.2 μW (right), respectively. f) PL spectra collected from the output channel, which is marked as “*O*_off_” for *P*_C_ = 0, and “*O*_on_” for *P*_C_ = 0.2 μW. The blue dashed line represents the threshold. Inset: The cyclic on/off switching behavior of the ports “*O*_on_” and “*O*_off_.” The white dashed lines in (b) and (e) are guides for eye to show the position of the structures.

A typical “H”‐like heterostructure with side and bridge lengths of about 60 and 40 μm, respectively (Figure 4b) was utilized to study the process of the excitons conversion inside. As displayed in the right panel of Figure 4b, the excitation with a 351 nm laser beam focused at the TPI nanowire can generate TPI excitons (Figure S5, Supporting Information). The excitons will diffuse along the TPI wire and transfer the energy to the energy acceptor ADN at the junction. Figure 4c shows the corresponding PL spectra collected from the junctions, which can be fitted by a superposition of TPI (405 nm) and (450 nm) spectra. This indicates that the propagated TPI excitons can be effectively converted to ADN excitons at the interface of the two kinds of materials.

The exciton conversion in the identical “H”‐like nanowire heterostructure, which can be regarded as an indirect modulation of output intensity, affords an approach to realizing photo­switch device for binary operation (Figure 4d). Two splitted laser beams were adopted to serve as input light and control light, respectively. The input beam was used to excite one of the ADN branches, and the output light signal was monitored from the end of the other ADN wire. The second laser beam was applied to pump the TPI nanowire, which plays the role of control signal for the modulation of the output signal. By varying the power of the control light, we can accurately modulate the output signal of ADN at the end via the synergy of the two excitations from input and control (Figure S6, Supporting Information). If we define a scattering intensity from the output terminal of >3000 as the “on” state, and the intensity <3000 as “off” state, the “H”‐like nanowire heterostructures would act as a photoswitch device.

As shown in Figure 4e, when a single input laser (I) was used to excite the ADN nanowire (Figure 4e, left), the output signal of ADN in white square is very weak (see the black line in Figure 4f), which is defined as the “off” state. When a control signal (*P*_C_ = 0.2 μW) was applied to excite the TPI nanowire (Figure 4e, right), the TPI excitons were generated at the excited position and propagated toward the heterojunction interface as EPs. At the junction region, the TPI excitons convert into ADN excitons, leading to the increase of the ADN signal. Therefore, a bright blue spot at the terminal can be observed, corresponding to the “on” state. The ADN intensity of the corresponding spectrum of *O*_on_ (the red line in Figure 4f) is nearly four times larger than that of *O*_off_. The relative ADN intensities for all the ten cycles almost remain unchanged (inset of Figure 4f), showing stable switching behavior with low device fatigue. We believe this concept can provide a novel approach to fabricate more complex structures for the manipulation of photons at subwavelength scale.

In conclusion, we report a cooperative molecular assembly strategy to construct “H”‐like organic nanowire heterojunctions with two parallel ADN nanowires bridged vertically by one TPI nanowire. By observing the growth process and theoretically calculating the binding energy of different molecules, the assembly mechanism of the binary molecular components was well explored. The TPI nanowires were formed first because the TPI–TPI molecules have higher binding energy; and then the two ends of the as formed each TPI nanowire act as nucleation centers for the site‐specific heteroepitaxial growth of ADN nanowires, resulting in the unique “H”‐like heterostructures. The effective exciton conversion at the junction enables the structure to be used as light‐controlled photoswitches for the manipulation of optical signals in miniaturized photonic devices. These results might provide enlightenment for the better understanding of molecular assembly toward specific structures and open up new prospects for the creation of novel photonic materials.

## Experimental Section

*Fabrication of Nanowire Heterojunctions*: The “H”‐like organic nanowire heterojunctions were prepared via a liquid phase cooperative self‐assembly method. In a typical preparation, 3 mL of hot (80 °C) ultrapure water as a poor solvent was dropwise added into 100 μL of the mixed stock solution of TPI (2 × 10^−3^ m) and ADN (5 × 10^−3^ m) in good solvent of methylene chloride. The rapid change of the surroundings induced the self‐assembly of TPI and ADN molecules. After cooling and aging in closed tubes at room temperature for 3 min, “H”‐like organic nanowire heterojunctions with uniform dimensions were obtained and dispersed in the colloid solutions. By changing the preparation conditions (e.g., temperature, TPI/ADN concentration ratios and aging time), the size of the heterostructures can be well tuned. The colloid solution was then used to prepare samples for further characterizations by drop casting onto silicon wafers and glass slides.

*Characterization*: The “H”‐like nanowire heterostructures were transferred onto different substrates for measurements by SEM (Hitachi, S‐4800), TEM (Jeol, 1011), and PL spectroscopy (Hitachi, F‐4500). The schematic demonstration of the experimental setup for optical characterization is shown in Figure S7 (Supporting Information). PL images were taken with an Olympus FluoView‐500 inverted microscope. To measure the PL spectra of single heterostructure, the sample were excited locally with a 351 nm argon ion laser (Spectra‐Physics, Beamlok2065, 150 nW) focused down to the diffraction limit. The excitation laser was filtered with a 351 nm notch filter. The light was subsequently coupled to a grating spectrometer (Acton SP‐2358) and recorded by a thermal‐electrically cooled CCD (Princeton Instruments, ProEm: 1600B). PL microscopy images were taken with an inverted microscope (Nikon, Ti‐U).

*Calculation Method*: All dimers were optimized at a hybrid density functional theory (B3LYP) level with the 6‐31 G (d,p) basis set utilizing the Gaussian09 d01 program.^18^ The 6‐31+G (d,p) basis set was used to obtain the single point energies on the optimized geometries. The binding energy for each dimer was corrected with the basis set superposition error.^19^

## Supporting information

As a service to our authors and readers, this journal provides supporting information supplied by the authors. Such materials are peer reviewed and may be re‐organized for online delivery, but are not copy‐edited or typeset. Technical support issues arising from supporting information (other than missing files) should be addressed to the authors.

Supplementary

Supplementary
