# Supplementary material for: “H”‐like Organic Nanowire Heterojunctions Constructed from Cooperative Molecular Assembly for Photonic Applications
Source: Adv Sci (Weinh). 2015 Jul 14;2(11):1500130. doi: 10.1002/advs.201500130 (PMC5115343; doi:10.1002/advs.201500130)
Supplement: Supplementary file 1 — Supplementary [file ADVS-2-0b-s001.pdf]

Copyright WILEY-VCH Verlag GmbH & Co. KGaA, 69469 Weinheim, Germany, 2015.

ADVANCED SCIENCE
Open Access

Supporting Information

for *Adv. Sci.*, DOI: 10.1002/advs. 201500130

"H"-like Organic Nanowire Heterojunctions Constructed from Cooperative Molecular Assembly for Photonic Applications

*Wei Yao, Guangchao Han, Fu Huang, Manman Chu, Qian Peng, Fengqin Hu, Yuanping Yi, Hua Jiang, Jiannian Yao, and Yong Sheng Zhao**

Supporting Information

'H'-like Organic Nanowire Heterojunctions Constructed from Cooperative Molecular Assembly for Photonic Applications

*Wei Yao, Guangchao Han, Fu Huang, Manman Chu, Qian Peng, Fengqin Hu, Yuanping Yi, Hua Jiang, Jiannian Yao and Yong Sheng Zhao**

Beijing National Laboratory for Molecular Sciences (BNLMS), Institute of Chemistry, Chinese Academy of Sciences, Beijing 100190, China, and College of Chemistry, Beijing Normal University, Beijing 100875, China

E-mail: yszhao@iccas.ac.cn

Contents

i. **Figure S1.** SEM images of ADN and TPI nanostructures.

ii. **Figure S2.** Fluorescence spectrum of TPI and absorption spectrum of ADN in solution.

iii. **Figure S3.** Bright-field optical microscopy images of TPI and ADN heterostructures prepared under different conditions.

iv. **Figure S4.** Molecular packing arrangements of TPI and ADN molecules at the interface of the ‘H’ like heterojunctions.

v. **Figure S5.** PL spectrum measured at the excited position on the bridge nanowire of an ‘H’-like heterostructure.

vi. **Figure S6.** The modulation behavior of the output signal by varying the power of the control signal.

vii. **Figure S7.** Schematic illustration of the experimental setup for the optical measurements.

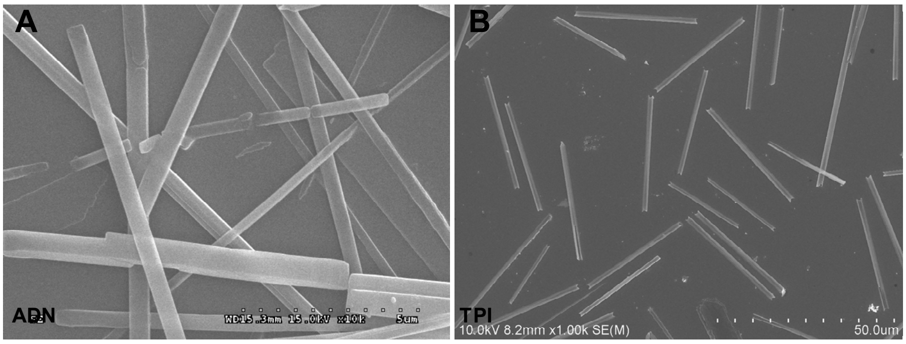

Figure S1. SEM images of ADN (A) and TPI (B) nanostructures.

Both ADN and TPI molecules can separately aggregate into homogeneous 1D nanowires with smooth surface and uniform diameter in liquid phase.

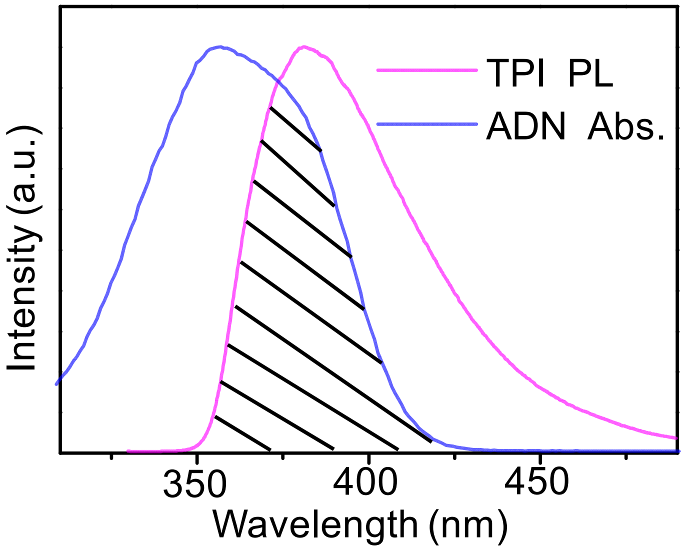

Figure S2. Fluorescence spectrum of TPI and absorption spectrum of ADN.

The fluorescence spectrum of TPI and absorption spectrum of ADN have a good overlap. This spectral overlap ensures the efficient energy transfer from TPI to ADN, which is very important for the tunable emission in the binary microstructures.

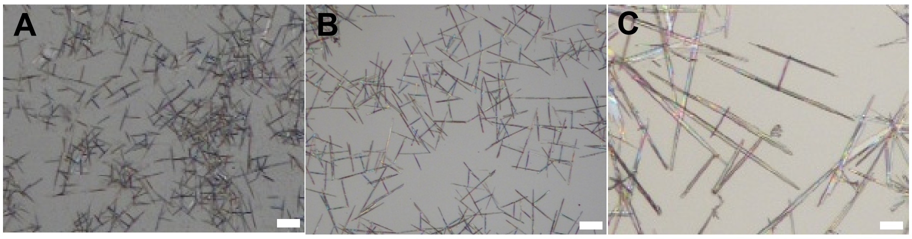

Figure S3. Bright-field optical microscopy images of TPI and ADN heterostructures obtained at different TPI/ADN concentration ratios of (A) 2:5, (B) 1:5, and (C) 1:10 with constant concentration of TPI (2 mM). All scale bars are 20 μm.

From the images we can see that the size of the heterostructures can be changed from 20 to 130 μm with the increase of ADN concentration, providing a flexible way to tailor the geometric structures of the 'H'-like nanowire heterojunctions.

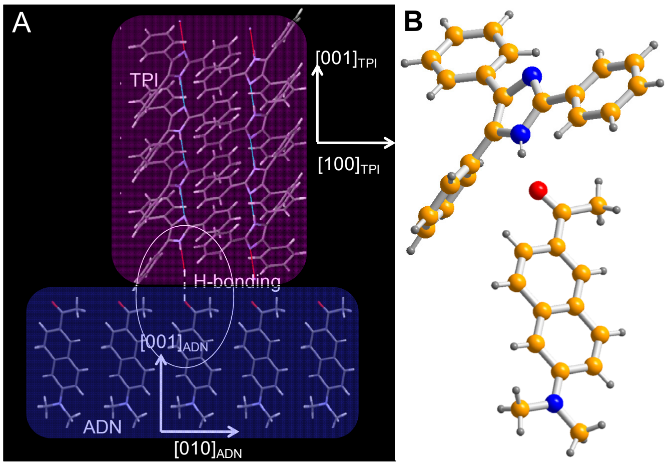

Figure S4. (A) Molecular packing arrangements of TPI and ADN molecules at the interface of the 'H' like heterojunctions. (B) High-magnification of the junction region marked with white circle in (A).

According to the growth direction of ADN and TPI in the nanowire heterojunctions, we can confirm the molecular arrangement at the junction region. As shown in Figure S4A, the ADN molecules are arranged in slip-stacks along the [010] directions, while two parallel chains of TPI molecules interdigitated with each other stack along the [001] direction via hydrogen bonding (-NH···N-). Based on this, we can construct the TPI-ADN molecular organization configurations (Figure S4B) to analyze the binding energy between them.

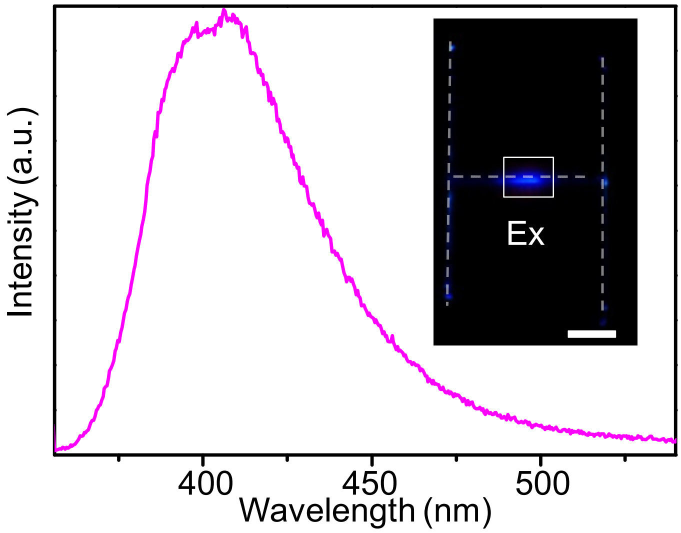

Figure S5. PL spectra spectrum measured at the excited position on the bridge wire of an 'H'-like heterostructure. Inset: PL image of the measured heterostructure locally excited from the middle of the TPI wire. Scale bar is 10 μm.

The PL spectrum of the excited point is in accordance with that of the TPI nanowires, which confirms that there are only TPI excitons generated from the excited position. In comparison, the PL spectrum shown in Figure 4c shows that the AND can be effectively excited at the interface of the junction. This indicates that the propagated TPI excitons can be effectively converted to ADN excitons at the interface of the two kinds of materials.

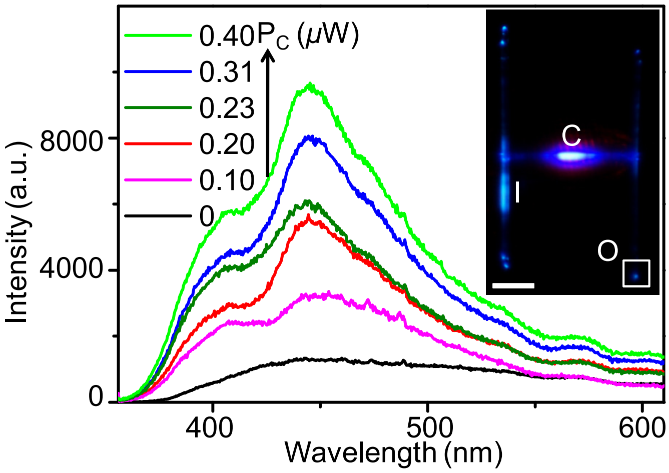

Figure S6. The PL spectra collected from the output channel (O) under a constant input laser power (P_I=0.2 μW) and varied control power from 0 to 0.4 μW. Inset: PL images of the measured heterostructure simultaneously excited with an input light (I) and control light (C). Scale bar is 10 μm.

With the increase of the control power, more TPI excitons were generated at the excited position and propagated towards the heterojunction interface, resulting in the enhancement of ADN signal at the output channel. This indicates that we can accurately modulate the output signal of ADN to realize a light-controlled photoswitch.

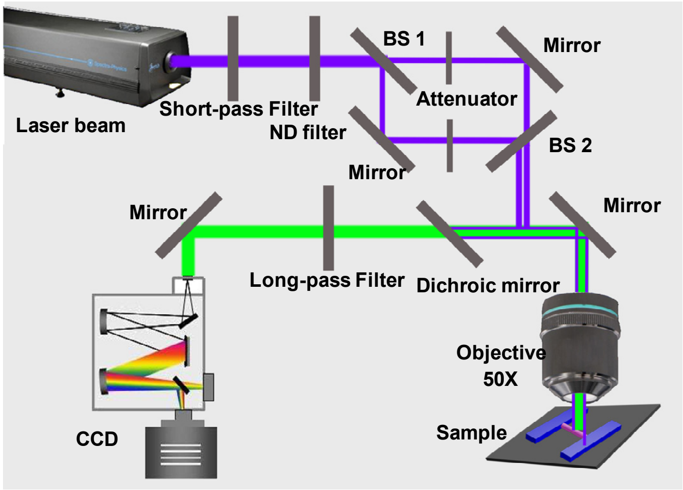

Figure S7. Schematic illustration of the experimental setup for optical measurements.

To measure the PL spectra of the nanowire heterostructures, the samples were excited locally with a 351 nm Argon laser (Spectra-Physics, Beamlok2065) focused down to the diffraction limit through an objective (Nikon CFLU Plan, 50×, N.A. = 0.8). The power at the input was altered by the neutral density filters. The emissions from the junctions of the heterostructures were dispersed with a grating (150 G/mm) and recorded with a thermal-electrically cooled CCD (Princeton Instruments, ProEm: 1600B).
